# The Origin of Translation: Bridging the Nucleotides and Peptides

**DOI:** 10.3390/ijms24010197

**Published:** 2022-12-22

**Authors:** Xuyuan Guo, Meng Su

**Affiliations:** 1 School of Genetics and Microbiology, Trinity College Dublin, The University of Dublin, College Green, Dublin 2, D02 PN40 Dublin, Ireland; 2 MRC Laboratory of Molecular Biology, Cambridge CB2 0QH, UK

**Keywords:** RNA world, transfer RNA, ribosome, peptidyl transfer, origin of life

## Abstract

Extant biology uses RNA to record genetic information and proteins to execute biochemical functions. Nucleotides are translated into amino acids via transfer RNA in the central dogma. tRNA is essential in translation as it connects the codon and the cognate amino acid. To reveal how the translation emerged in the prebiotic context, we start with the structure and dissection of tRNA, followed by the theory and hypothesis of tRNA and amino acid recognition. Last, we review how amino acids assemble on the tRNA and further form peptides. Understanding the origin of life will also promote our knowledge of artificial living systems.

## 1. Introduction

Among the three steps in the central dogma, translation is the most important as it bridges the world of nucleic acids and the world of amino acids. In the “RNA world” hypothesis [1], RNA came first from the primordial environment, recorded the genetic information, and catalyzed fundamental biochemical reactions. Later, RNA alienated the catalytic function of peptides and proteins and released the information storage function to DNA. In general, peptides are more diverse in structure and DNA is more stable than its RNA counterparts.

DNA self-copy, i.e., replication, and DNA-templated RNA polymerization, i.e., transcription, are more intuitive and practicable in prebiotic settings compared to RNA-coded peptide formation. Nowadays, translation takes place in the endoplasmic reticulum membrane in the cytoplasm with messenger RNA as the template, transfer RNA as the adaptor, and ribosome RNA as the catalytic core. How tRNA and mRNA formed and how primal peptides were coded in the prebiotic era without auxiliary proteins are interesting questions. Although these questions were posted decades ago and various theories have been put forward, great attention and practical effort have been given to these issues in recent years. Here, we summarize the hypotheses and experimental milestones regarding this question. We focus on tRNA evolution, codon assignment, and coded peptide formation, and provide our ideas on the origin of translation.

## 2. The Evolution of tRNA

**Figure 1:**
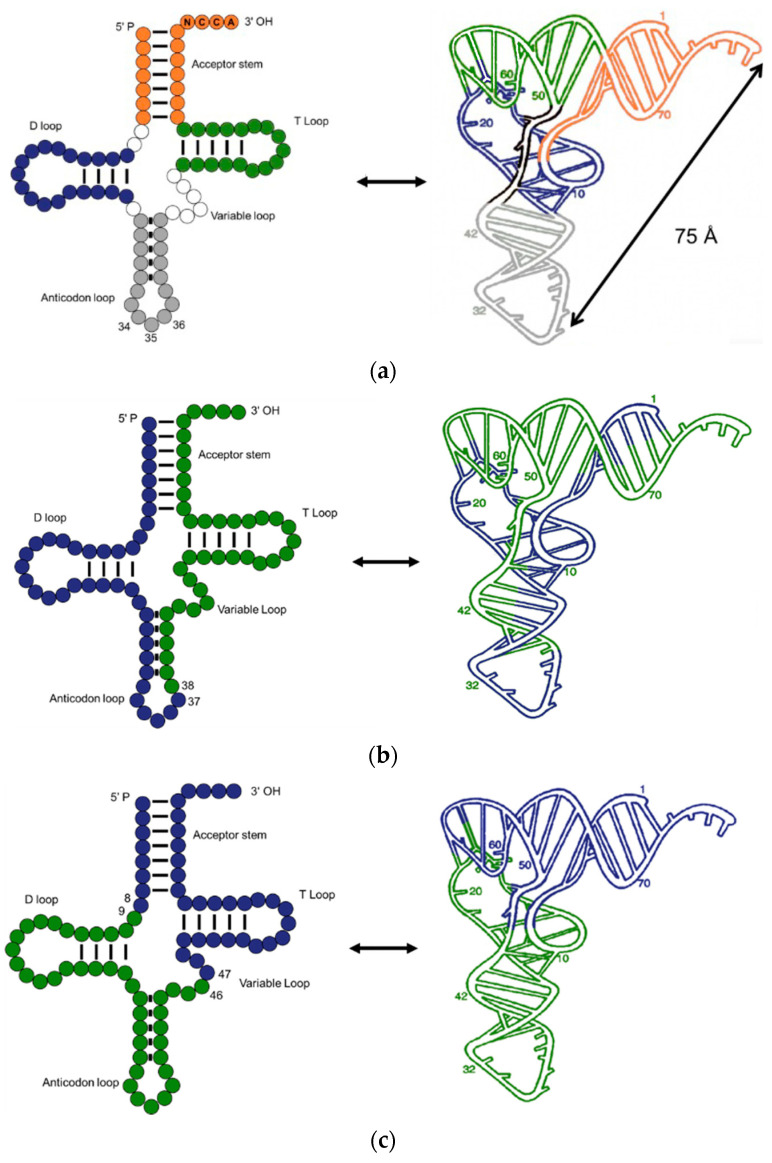
**Structure and dissections of a type I tRNA from *E. coli*.** (**a**) Typical cloverleaf 2D and ribbon diagram 3D structure, different loops and stem are colored accordingly; (**b**) dissection at anticodon loop; (**c**) dissection at the acceptor domain and anticodon domain.

Transfer RNA, the core of genetic coding, is an invaluable molecular fossil that can help in discovering the origin of translation. After revealing the double helix structure of DNA in 1953, Crick [2] proposed that an adaptor-like RNA molecule connected the DNA and proteins. This adaptor-like RNA was then proved by Hoagland et al. [3] and named transfer RNA. Nowadays, our knowledge of tRNA has increased dramatically. A typical tRNA is made up of 76 nucleotides, but the numbers can be 72–96. The classical secondary structure, usually called a “cloverleaf” structure, consists of a CCA end (position 74–76), acceptor stem (1–7 and 67–73), anticodon loop (30–46), T-loop (52–68), D-loop (8–24), and variable loop (Figure 1a). Although most tRNAs show the classic structure, tRNAs without T-loop, D-loop, or a double helix acceptor stem were identified in the mitochondria of several metazoans [4,5,6]. When folded up, tRNA adopts a near vertical angle geometry; where the CCA end is 75 angstroms away from the anticodon loop. Magnesium ions play a role in stabilizing the tRNA geometry [7]. 

In extant life, the DNA sequence that codes the tRNA is either separated with introns, fragmented, or rearranged [8]. This is probably the result of the co-evolution of tRNA and RNA splicing endonuclease [9]. Split and fragmentation in tRNA genes are deemed late acquisitions [10] or a vestige of early tRNA [11]. In human cells, tRNA is the most abundant RNA among all cellular RNAs and is most intensively modified. The human genome contains more than 400 tRNA genes to decode 61 codons [12,13], i.e., there are multiple tRNA genes and tRNAs carrying identical anticodon sequences for one specific cognate amino acid. tRNA with the same anticodon but are called ‘isodecoders’ while tRNA with different anticodon but charged with the same amino acids are called ‘isoacceptors’. Cellular and mitochondrial tRNA overexpression and mutation relate to a wide range of human diseases [14,15,16] such as breast cancer [17] and neurogastrointestinal encephalopathy [18].

**Figure 2:**
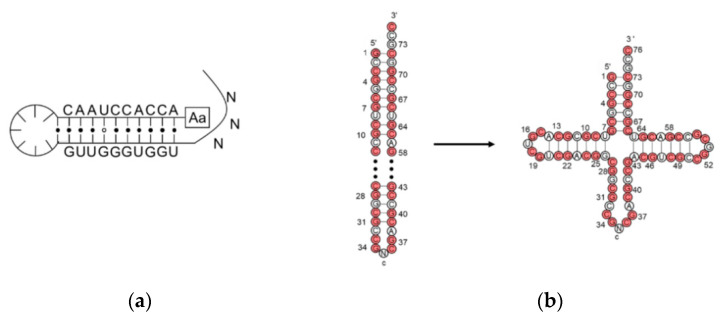

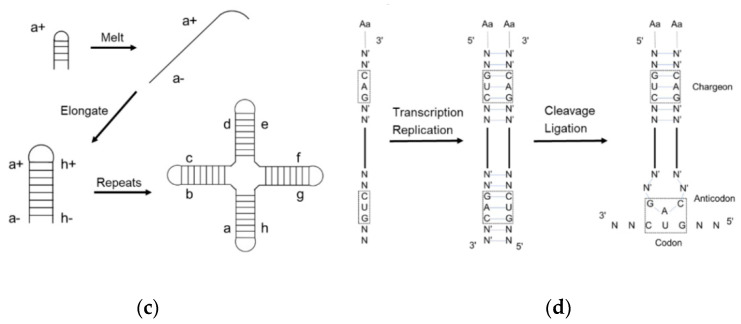
**Early models of tRNA evolution.** (**a**) Hopfield model showing trinucleotide interaction with the amino acid at 5′-terminus; (**b**) Winkler-Oswatitsch and Eigen model showing how tRNA came from RNY-type triplets; (**c**) Bloch model showing how tRNA came from RNA self-priming and self-templating; (**d**) Moller model showing how tRNA came from strand replication and ligation.

Past decades have witnessed significant progress focusing on the stepwise assembly of tRNA. In the beginning, Woese [19] posited that a subunit of tRNA emerged first and executed a partial role of the extant tRNA. Then, Hopfield [20] suggested that the first primitive tRNA was an RNA hairpin with an amino acid at the 5′-terminus and a foldback overhang on the 3′-terminus (Figure 2a). The overhang was supposed to interact with and recognize the amino acids. It is a reasonable idea that the hairpin was the most abundant RNA secondary structure in the prebiotic era due to the replication of a single-stranded RNA using itself as the template. However, in the extant tRNA, the anticodon loop is far away from the acceptor stem and any physical interaction is impossible. Although tri-loops are common secondary structure motifs found in naturally occurring RNA [21], the direct interaction of loop would not be limited to a trinucleotide in Hopfield’s model. More nucleobases can be included. Later, Winkler-Oswatitsch and Eigen [22] proposed that the tRNA was made up of a 76-mer hairpin which further assembled the cloverleaf shape tRNA (Figure 2b). A 39-mer strand, i.e., thirteen RNY-type (purine-N-pyrimidine) triplet nucleotides, acted as the template for the counter strand to form a 78-mer hairpin. Both the Hopfield and Winkler-Oswatitsch models recognized the hairpin as the precursor of tRNA, but the latter model suggested that a long hairpin directly evolved to the extant geometry. Bloch and coworkers [23] postulated a self-priming and self-templating model explaining the formation of tRNA and rRNA (Figure 2c). A cruciform RNA was generated after three cycles of self-replication and denaturation. The final product is perfectly symmetrical, while the extant tRNA is not. Therefore, it was assumed that the final structure could not be achieved via perfect direct duplication. Moller and Janssen [24] suggested a progressive tRNA evolution model, interpreting the anticodon triplets transited from the acceptor stem to the extant anticodon loop (Figure 2d). The tRNA evolution consisted of three steps: transcription, cleavage, and ligation. Different from the Winkler-Oswatitsch model, Möller thought the tRNA came up from two complementary single-stranded RNA followed by loop ligation of the 5′-end with the 3′-end of the opposing strand to yield the anticodon loop. However, prebiotic possible ligation chemistry was not achieved until recently [25], and correlations between the anticodon triplet and the “chargeron” in the acceptor stem cannot be found in the extant tRNA. 

**Figure 3:**
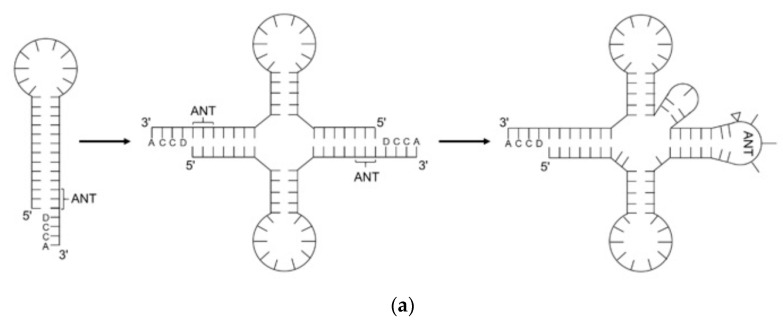

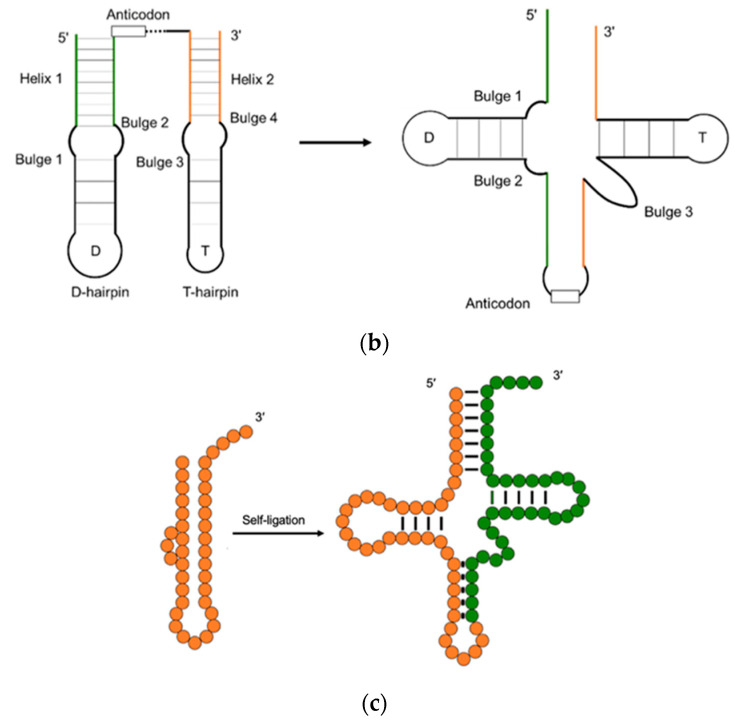
**Recent models of tRNA evolution.** (**a**) Di Giulio model showing how tRNA came from homodimer hairpins; (**b**) Tanaka model showing how tRNA came from two distinct hairpins with bulges; (**c**) Fox model showing how tRNA came from two identical hairpins with bulges by self-ligation. ANT, anticodon. D, base-determinator.

At the same time, Di Giulio [26,27] modified the hairpin model by suggesting that the tRNA arose from a hairpin homodimer. Two ends of hairpins were then ligated to yield the extant tRNA (Figure 3a). Phylogenetic analyses demonstrated that hairpins duplicated prior to the divergence of three domains of life [28]. Inspired by the Giulio model, Tanaka et al. [29] proposed a model illustrating that tRNA originates from two distinct hairpins, corresponding to extant D- and T-loop. The hairpins were stabilized by base modification, intron, and bulges (Figure 3b). Conjugating the two hairpins was the intermediate step prior to the cloverleaf structure. The double hairpin intermediate model is more plausible as it explained the origin of tRNA introns. In contrast, Nagaswamy et al. [30] pointed out that the tRNA might have risen by ligating two identical 38-mer hairpins. The extant tRNA structure can be configured by ligation of the two hairpins with a bulge at the 8–10 position and an NCCA end (Figure 3c). The common ground in the Giulio, Tanaka, and Fox models is that two concurrent hairpins are ligated at the anticodon loop (Figure 1b). Denaturing and reannealing of the hairpins followed by ligation lead to the extant tRNA. However, the selection pressure of specific hairpins is not clear and the thermodynamic benefits of recombining two denatured hairpins to the L-shaped geometry are not guaranteed.

Contrary to the above models, Maizels and Weiner [31] disassembled the tRNA into two domains, i.e., a conserved top half domain and a non-conserved bottom half domain, connected at the positions 8/9 and 46/47 (Figure 1c). The top half of the extant tRNA is recognized by aminoacyl tRNA synthetases (aaRS), rRNA, RNase P, and EF-Tu, while the bottom part is an independent hairpin which was incorporated later. Crystallographic studies support the model showing that the energy consumed for rotation of the two halves is associated with the swing angles [32]. The two halves are highly flexible as the energy cost for the swing is low. Such flexibility facilitates interactions between tRNA and other macromolecules. The acceptor stem domain is older than the anticodon loop domain [33]. Following the top and bottom halves idea, William et al. [34] further put forward the accretion model, which explained the origin and evolution of the translation machine based on spatial comparative analysis. The evolution of the translation system was divided into six accretion phases, while tRNA assembly was completed in the first three phases. In the first phase, the tRNA is an oligonucleotide with a CCA end. tRNA gained the acceptor stem as well as the T loop to form a minihelix in the second phase. In the third phase, the minihelix was expanded by insertion to form an L-shaped geometry that fulfilled the bi-functional requirements in extant tRNA. Furthermore, the L-shaped tRNA interacts with the small ribosomal subunit (SSU) and large ribosomal subunit (LSU) and forms a noncovalent quaternary complex together with proto-mRNAs. The double hairpin model and the two halves model agree that two parts of tRNA were segregated at first but diverged in the ligation position and on the chronological order of the two parts. 

Several further ideas have been put forward along with the models above, including the tri-minihelices model [35,36], the circular tRNA model [37,38], and the Fibonacci-like model [39]. Debates between these models are inevitable [40]. However, the strongest evidence that supports a hypothesis always comes from wet-lab experiments, not from deduction.

## 3. Assignment of the Amino Acids to Genetic Codes

Before discussing the onset of RNA-coded peptides, the emergence of RNA and amino acid precursors in the prebiotic settings needs to be clarified. In the Miller experiment, various products were synthesized and identified under a possible primitive condition. Gly, Ala, and β-Ala were identified at the 100 µM scale. Asp, Glu, Ser, and Val were observed as well, albeit in a lower concentration [41,42,43]. Later, prebiotic chemistry demonstrated a unified network of nucleic acids, amino acids, and lipids [44]. Precursors of RNA and amino acids were generated by the reductive homologation of hydrogen cyanide. Apart from the amino acids mentioned above, Pro, Leu, Ile, Thr, and Arg might also arise from the primordial soup. Interestingly, most prebiotic possible amino acids are assigned to the four-degenerated codons, or family boxes, proposed by Lagerkvist [45], while the latecomers are assigned to the two-degenerated codons, or split boxes, where purine or pyrimidine at the third position of the triplet make a difference in the assignment. This indicates that four-degenerated codons were first occupied and used in the mRNA. Initial peptides may only consist of prebiotic amino acids. In vitro and in silico experiments have shown that prebiotic amino acids alone are capable of yielding soluble and foldable proteins with various secondary structures, nucleophilicity, and metal binding ability [46,47]. On the other side, the universal set of amino acids is a comprehensive assessment of biosynthetic cost, solubility, stability, etc. [48]. A consensus chronology of amino acids has been built based on many different criteria [49,50].

Due to the sequential emergence of amino acids, the assignment of genetic codes must be stepwise. The genetic codes are universal in the three kingdoms; therefore, the assignment of amino acids to the 64 triplets must be established before the division of the kingdoms. In parallel with the evolution of tRNA, several hypotheses for codon assignment have been proposed in the past decades, among which the frozen accident theory and stereochemical theory were first. Frozen accident theory claimed that codons were randomly assigned and are impossible to change significantly afterwards [2,51]. The introduction of a new correlation between an amino acid and a codon triplet occurs only if such mutation benefits fitness. However, the frozen accident theory did not solve the puzzle satisfactorily. The degeneracy of the genetic code and the stability of the second base pair of anticodons revealed that the evolution of assignment was accompanied by a single base changing [52]. A simple structural change in an amino acid is always accompanied by a changing of one nucleotide in the triplet, usually the third codon [53], e.g., Asp (GAY) and Glu (GAR), Val (GUN) and Leu (CUN/UUR). Thus, it is unlikely that the amino acid’s assignment is the result of a merely random combination. 

Stereochemical theory proposed a different viewpoint: that the assignment was rational because of the stereochemical interactions, or the physical affinity, between the amino acids and nucleotides. The initial attempt to interpret the interaction, which dated back to 1954, was the diamond code theory, which proposed that a ‘key-and-lock’ relationship existed between a specific amino acid and the rhomb-shaped ‘holes’ in the DNA strand [54]. The diamond code theory was further developed as the stereochemical theory and numerous models relating to this have since been proposed [55,56,57,58,59]. Furthermore, a direct correlation has been observed between the hydrophobicity and the hydrophilicity of the amino acids and their anticodon nucleotides [60,61]. Recent crystallographic evidence seems to support the stereochemical theory. Anticodon triplets in ribosome RNA are enriched close to their cognate amino acids in the r-proteins [62]. Most amino acids with significant anticodon enrichment were not observed in the Miller experiments, leading to the deduction that only late amino acids’ assignment is involved in anticodon interactions. As is known, rRNA is the core for catalyzing peptidyl transfer and the r-proteins emerged much later. Late amino acids in r-proteins may indeed interact with the nucleotides in the rRNA; however, how the initial prebiotic amino acids were assigned is still unsolved. 

The G·U wobble base pair in the acceptor stem supports the stereochemical theory. The G3·U70 determines the specificity of the tRNA selection of Ala throughout the evolution from bacteria to humans [63]. Mutating the G·U pair in *E. coli* tRNA^Ala^ eliminated aminoacylation both in vivo and in vitro [64]. Comparisons between A3·U70 and G3·U70 further confirmed that the wobble base pair is the identity element of tRNA^Ala^ as the A3·U70 folded back into a non-reactive conformation [65]. Thus, the G·U wobble pair was assumed to be the identity element of tRNA^Ala^ until recently [66], indicating that there are physical interactions between the tRNA acceptor stem and the alanine at the 3′-terminus. Recent experiments suggest that the acceptor stem codes for the size and branched and carboxylic acid sidechains that facilitate the production of antiparallel peptides, making the stereochemical theory more plausible [67]. 

As neither the frozen accident theory nor the stereochemical theory provide a comprehensive solution to the assignment of the genetic codes, further theories have been put forward. The co-evolution theory addressed the issue by asserting that the extant codon assignments were defined according to the sequential emergence of amino acids [68]. The codon assignment is the vestige of the prebiotic amino acid synthesis, which remains in the extant amino acids biosynthesis pathways. Development of the genetic code could be deduced from the precursor–product connections among the amino acids. As the precursors evolved into extant amino acids, co-evolution among amino acid precursors, amino acids, and aaRS took place [69,70]. For example, Trp is considered a later addition to the genetic code owing to TyrRS divergence and neofunctionalization [66,71,72].

Translation error theory claims that evolution is aimed at reducing errors in translation. Coding started from random assignment and codons for chemically related amino acids were adjacent in the codon table, resulting in high robustness against RNA mutations and translation errors [73]. Ambiguous codon assignments with high entropy binding were progressively replaced with lower binding entropy, suggesting that the extant genetic code emerged from the codes with a lower level of certainty. The certainty increased along with tRNA species expanding during their evolution [74]. The codon assignment reduced base-pairing errors and phenotypic impacts from mutations to the minimum level. Single base alterations, particularly transitions, generally result in no or conservative amino acid replacement. The GC content of amino acid codons is related to the degree of codon degeneration. Third-position degeneration and high GC content provided an additional layer of protection for the primary amino acids that constructed preliminary peptides. Such codon assignment provides increased systematic perseverance for protein synthesis [75]. The fitness of the current codes has been settled by the growing robustness of the translation machine that originated from the initial codon assignment. 

The above theories for codon assignment are not mutually exclusive [76,77]. A hybridized theory would be an option for codon assignment. First, physical interactions between the prebiotic amino acids subset and short RNA strands, similar to the extant acceptor stem, affected the esterification at the 3′-terminus of the RNA (which will be discussed in the next section). The interaction could have been positive, i.e., ribose aminoacyl ester formed where there was a strong interaction between the amino acid side chains and nucleotides, or it could have been negative, i.e., aminoacyl esters formed where the interaction was weak. Even though the selectivity of the amino acid to a trinucleotide is low, the preference would manifest and magnify over time. Next, the tRNA acceptor stem domain and the anticodon loop domain merged randomly and froze thereafter, which is the same as the Maizels and Weiner model. At this stage, chemically alike amino acids could not be differentiated. The minor alterations in peptides were allowed by inducing mutations in mRNA. Peptidyl RNA esters and the corresponding hydrolyzed products (peptides) could be functional and benefit the essential biochemical processes, e.g., nucleotide ligation and cleavage, peptidyl ester transfer and hydrolysis, and peptide folding. This step may have lasted for a length of time before aromatic and sulfur-containing amino acids emerged. Lastly, aaRS co-evolution and adaptation enabled canonical amino acid assignments that aimed at more fitness for survival.

## 4. Coded Peptide Formation

Dipeptides can be produced through the amidation of two amino acids without coupling reagents, but the yield is low [78]. The amino acid activation under primitive conditions with various coupling methods has been recently reviewed [79]. Typical inorganic catalysts include layered double hydroxides [80] and titanium dioxide [81]. Cyanamide, diaminomaleonitrile, ferricyanide, trimetaphosphate, etc. are organic activators that likely catalyze the amide bond formation under prebiotic conditions. Prebiotically plausible N-carbamoyl amino acids (NCA) [82], 5(*4H*)-oxazolone [83,84], and cyclic acylphosphoramidate (CAPA) [85], activated from the respective amino acids, further yield mixed anhydrides and aminoacyl esters. Although prebiotic peptide formation is prevalent and short random peptides can be functional, the function cannot self-propagate until the amino acid sequence is recorded in RNA or prebiotic nucleotide analogues [86].

**Figure 4:**
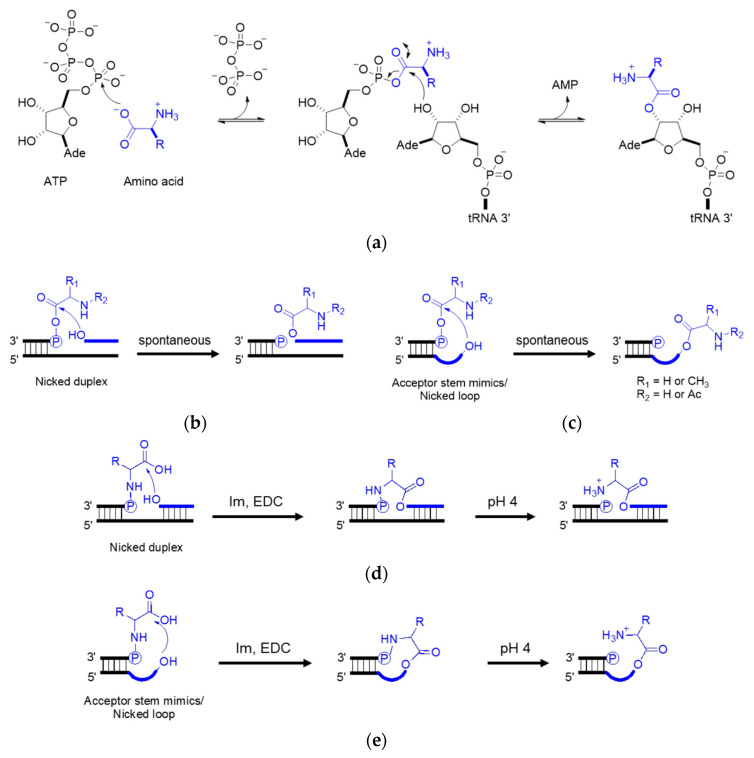
**Aminoacyl esterification on the RNA 3′ terminus.** (**a**) Amino acid activation using aaRS in the extant biology; (**b**) aminoacyl transfer in a nicked duplex; (**c**) aminoacyl transfer in a nicked loop; (**d**) phosphoramidate-mediated esterification in a nicked duplex; (**e**) phosphoramidate-mediated esterification in a nicked loop. Im, imidazole. EDC, 1-ethyl-3-(3-dimethylaminopropyl) carbodiimide. R, amino acid residue.

In the extant biology, peptide synthesis starts with the acylation of the 3′-terminus of the tRNA using aaRS (Figure 4a). In the prebiotic context, acylating RNA strands without the presence of pre-synthesized enzymes initiates RNA-coded peptide synthesis [87]. Tamura et al. [88] and Wu et al. [89] discovered that aminoacyl spontaneously transferred from aminoacyl phosphate mixed anhydride at the 5′-terminus of a donor strand to the 3′-terminus of an acceptor strand in a nicked duplex (Figure 4b) or nicked loop (Figure 4c). The transfer showed good stereoselectivity of L- over D-amino acids and chemical selectivity among amino acids, which indicates that the conformation of the D-ribose and β-nucleoside resulted in the single-chirality of amino acids. In the case of the nicked duplex transfer, tri-phenylalanine-RNA ester was detected [90]. Similar esterification was achieved via phosphoramidate (Figure 4d,e) [91,92]. While the prebiotic synthesis of mixed anhydride is not yet solved, amino acid phosphoramidate is prebiotic accessible [61]. To achieve the RNA-coded peptide synthesis, either the formation of amino acid donors or the aminoacyl transfer should be processed in an RNA sequence-dependent manner; however, no sequence-dependent acylation has been reported to date.

Research has been carried out to search for primordial evidence for aminoacyl-RNA catalyzed using ribozymes. The initial efforts involved selecting an aptamer that could rapidly and specifically aminoacylate its 2′/3′-OH using phenylalanine adenylate [93,94]. Further deducting the ribozyme to five nucleotides retained the acylation activity [95]. Up to penta-phenylalanine peptide was detected using the pentamer ribozyme. Although the structure has proven its capability to undertake the function of extant aaRS, its dissimilarity from the tRNA minihelix domain and the prebiotic inaccessible phenylalanine adenylate means it is not relevant to prebiotics. Moreover, as the relation between the RNA and amino acids was not illustrated, this strategy cannot be deemed as RNA-coded peptide synthesis, even though polyphenylalanine was identified.

**Figure 5:**
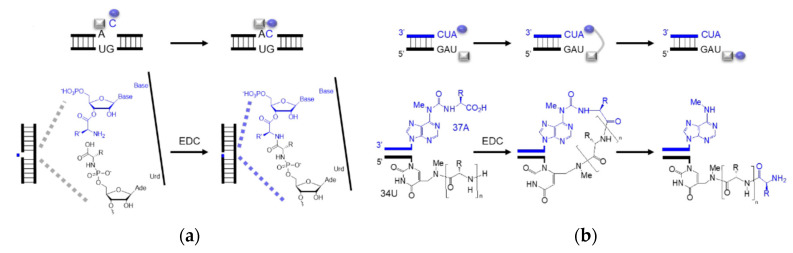
**Sequence-dependent amino acid coupling.** Schematic representation of (**a**) ‘mononucleotide translation’ in a nicked duplex using one ssRNA as a template and two complementary RNAs with aminoacyl ester and phosphoramidate; (**b**) peptide formation in a tRNA anticodon loop mimic using m^6^aa^6^A and mnm^5^U. Rectangle and oval shapes represent amino acids. Bold lines represent RNA strands.

Enzyme-free RNA-templated peptide synthesis has been reported using 5′-phosphoramidate amino acids (Figure 5a) [96] and amino acids conjugated to modified RNA bases (Figure 5b) [97]. Amidation selectivity has been observed between the nucleoside monophosphate and the nicked duplex, validating the idea of ‘mononucleotide translation’. The mononucleotide translation is believed to evolve to the extant triplet code system when adapted to a higher diversity of amino acids. Parallel to the nicked duplex, RNA hairpins, analogues to the tRNA anticodon loop, have also been seen as the platform of peptide synthesis. Non-canonical RNA bases, as well as peptide–RNA chimaera, are assumed as the vestiges of the RNA world that played a role in coded peptide synthesis [98]. In both scenarios, activators such as EDC, which is not considered prebiotic accessible, were used as coupling reagents while the extant biology utilizes aminoacyl ester transfer to form the amide bond. A more possible prebiotic scenario should be comparable to the extant mechanism. Additionally, if the nicked duplex or anticodon loop templated amidation were authentic, a transition of peptide formation from the nicked anticodon loop to the extant acceptor stem should be envisaged.

**Figure 6:**
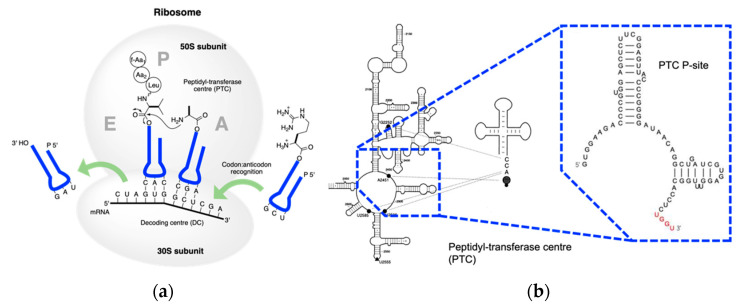
**Extant peptidyl transfer.** (**a**) Mechanism of peptidyl transfer and peptide elongation in the extant biology; (**b**) detailed structure of PTC in the LSU interacts with tRNA (adapted from [110]). G2252, U2506, and U2585 interact with the CCA end of the tRNA. The enlargement shows the P binding site. UGGU in red indicates an engineered handle for the CCA end in the tRNA.

The second step of translation is peptidyl transfer, which is assisted by the ribosome; this is one of the core processes in extant biology. In all three kingdoms of life, the ribosome catalyzes the peptidyl transfer and monitors the accuracy of the mRNA-templated translation. Dissection of ribosomes enlightens our understanding of translation in prebiotic times. The prokaryotic ribosome consists of two separate components, the SSU and the LSU (Figure 6a). The SSU contains a decoding centre (H44) while the LSU contains a peptidyl transferase centre (PTC) [99]. As LSU alone can catalyze peptide bond formation in vitro in the absence of the SSU [100], the LSU should have preceded the SSU in evolution.

LSU is divided into six domains based on structure and function, among which domain V accounts for peptidyl transferase activity [101]. Interaction between PTC and tRNA on the A-site is responsible for amino deprotonation, water proton transfer, and tetrahedral intermediate formation [102], while r-proteins frame the ribosome structure [103,104]. Therefore, Cech [105] had his wise saying ‘The ribosome is a ribozyme’. It is worth mentioning that apart from amidation, the ribosome and ribozymes could catalyze esterification when puromycin was replaced with hydroxypuromycin [106,107,108]. These examples indicate that the proto-ribosome may have evolved from hydroxylacyl transfer to the extant aminoacyl transfer. Thus, the RNA-coded depsipeptides and polyesters world is a plausible alternative to the RNA–peptide world [109].

Several mimicked or minimized ribosome PTCs have been designed and tested. A selected PTC-like ribozyme suggested that short, folded oligoribonucleotides could aid RNA–RNA interaction and peptidyl transfer, further proving the idea that the LSU originated from a short RNA [111]. Based on the symmetrical structure of PTC, Yonath et al. proposed that the PTC evolved by gene fusion. A semi-symmetrical pocket-like structure, which functioned as the proto-ribosome, was further partitioned into A-region and P-region according to the interaction with the A-site or P-site tRNA [112]. The pocket-like structure catalyzed the peptidyl transfer and supported the tRNA move from the A-region to the P-region [113]. A 9-mer polylysine peptide was observed when both regions were presented [114], but the characterization was vague. When testing A- and P- regions separately, analysis of the resulting dipeptide revealed that the P-region was responsible for catalyzing the amidation (Figure 6b) [115]. The yield of the dipeptide was reported to increase 4.2-fold when a UGGU handle was engineered to the P-region intended to promote the CCA-end proximity [116]. Outside the A-/P-regions, the structural analysis demonstrated that, in the PTC, two guanosines, G2251 and G2252, bind to P-site tRNA C74/75, and one guanosine, G2553, directly binds to A-site tRNA C75 [117]. The proximity of two tRNAs and the efficiency of the peptide bond formation are guaranteed through base complementarity [118]. Based on the above research, one can imagine that, in a Darwin Pond with activated amino acids and short RNA, some RNA was able to catalyze the peptidyl transfer within other aminoacyl-RNA, yielding the initial peptidyl-RNA and peptides. The remaining parts of the ribosome, e.g., the exit tunnel and proofreading centre, thrived over time.

## 5. Conclusions and Perception

We have reviewed here three major questions about the origin of translation: tRNA evolution, codon assignment, and coded peptide formation. The two halves model from Maizels and Weiner indicates that the acceptor stem domain is ancient and ligates with the anticodon loop domain. The frozen accident theory would be correct if the fusion of the two domains were random. The stereochemical theory would also be correct if sequence-dependent aminoacyl transfer was discovered. Coded peptide formation without previously synthesized peptides should have been possible when tRNAs were juxtaposed with an ssRNA with the help of ribozymes. Other topics of concern, e.g., amino acids activation, random peptide synthesis, etc., may also be interesting but are not covered here.

Numerous issues remain unsolved or have not even been investigated. To the best of our knowledge, no research has shown whether mRNA or PTC came first. If peptides can arise from anticodon loops and ssRNA complexes without PTC, then mRNA must have come earlier than the ribosome. The ssRNA, which acted as the extant mRNA, recorded the peptide sequence. To create positive feedback, the initial peptides must take on the function to promote the RNA loop and ssRNA interaction and accelerate peptidyl transfer. However, if a peptide could be formed without ssRNA but needs a ribozyme, then mRNA must come later than PTC or the proto-ribosome. Although the peptides could assist aminoacylation and peptidyl transfer, how could the sequence information in the peptide be recorded [119]? This puzzle is worth further study.

Extant prokaryotic translation starts with N-formylmethionine (fMet). The formyl group at the amino end of a dipeptide prevents it from cyclizing to 2,5-diketopiperazine (DKP), which is highly stable and cannot be linearized. Therefore, the formyl protecting group is a good strategy to preserve peptidyl transfer products; however, did the N-formyl amino acid present prior to acylation, or did free aminoacylated tRNA arise first then the formyl group came to cap the N-terminus? Did the chemistry for recycling DKP already exist in the Darwin Pond, or did the DKP never bother the peptide formation? This is currently unknown.

As is known, peptides can form spontaneously in the prebiotic context [120,121]. They can even display functions as short as dipeptides [122]. However, when and how were the random peptides taken over by the coded peptides? Did the coded peptide come much later than we expected, and were random peptides able to propagate themselves with sequence or conformation similarity, as in amyloids [123,124] and prions [125]? 

Compartmentalization is requisite for a living system. Did the compartment arise after the translation was established, or did they emerge at the same time? Would the compartment benefit the translation efficiency and fidelity? How did the translation products trigger the Darwinian evolution? These intriguing and challenging questions are left open for further study.

Investigating the origin of translation not only provides us with the knowledge of the origin of life and satisfies our curiosity about abiogenesis, but also enables us to build non-canonical and functional biopolymers in a cell-free medium or protocells. Knowledge of the translation machine, including the tRNA and rRNA, helps us to develop new therapies on the protein expression level. Recent advances in suppressing premature termination codons with anticodon-engineered tRNA are encouraging steps to conquer single-gene disorders using non-canonical tRNA [126,127,128,129]. Finally, thanks to the availability of X-ray crystallography, cryo-EM, sequencing methods, and advanced analytic tools, we have gained knowledge on the translation machine over the past decades; however, the origin of translation is still a chemical and biological puzzle. We have much more to learn to bridge the nucleic acids and amino acids and to illustrate the translation at the molecular level.

## Data Availability

Not applicable.
